# Combined Carbohydrates Support Rich Communities of Particle-Associated Marine Bacterioplankton

**DOI:** 10.3389/fmicb.2017.00065

**Published:** 2017-01-31

**Authors:** Martin Sperling, Judith Piontek, Anja Engel, Karen H. Wiltshire, Jutta Niggemann, Gunnar Gerdts, Antje Wichels

**Affiliations:** ^1^Alfred-Wegener-Institute Helmholtz Centre for Polar and Marine ResearchBremerhaven, Germany; ^2^Biologische Anstalt Helgoland, Alfred-Wegener-Institute Helmholtz-Center for Polar and Marine ResearchHelgoland, Germany; ^3^Biological Oceanography, Marine Biogeochemistry, GEOMAR Helmholtz Centre for Ocean Research KielKiel, Germany; ^4^Research Group for Marine Geochemistry (ICBM-MPI Bridging Group), Institute for Chemistry and Biology of the Marine Environment, University of OldenburgOldenburg, Germany

**Keywords:** bacterioplankton community, remineralization, extracellular enzymes, phytoplankton bloom, North Sea, Helgoland Roads, organic matter, spring bloom

## Abstract

Carbohydrates represent an important fraction of labile and semi-labile marine organic matter that is mainly comprised of exopolymeric substances derived from phytoplankton exudation and decay. This study investigates the composition of total combined carbohydrates (tCCHO; >1 kDa) and the community development of free-living (0.2–3 μm) and particle-associated (PA) (3–10 μm) bacterioplankton during a spring phytoplankton bloom in the southern North Sea. Furthermore, rates were determined for the extracellular enzymatic hydrolysis that catalyzes the initial step in bacterial organic matter remineralization. Concentrations of tCCHO greatly increased during bloom development, while the composition showed only minor changes over time. The combined concentration of glucose, galactose, fucose, rhamnose, galactosamine, glucosamine, and glucuronic acid in tCCHO was a significant factor shaping the community composition of the PA bacteria. The richness of PA bacteria greatly increased in the post-bloom phase. At the same time, the increase in extracellular β-glucosidase activity was sufficient to explain the observed decrease in tCCHO, indicating the efficient utilization of carbohydrates by the bacterioplankton community during the post-bloom phase. Our results suggest that carbohydrate concentration and composition are important factors in the multifactorial environmental control of bacterioplankton succession and the enzymatic hydrolysis of organic matter during phytoplankton blooms.

## Introduction

It is well established that marine bacterioplankton communities maintain high genetic diversity. As for phytoplankton (Hutchinson, 1961), little is understood, however, how such high diversities are maintained or what determines the community composition *in situ*. In seasonal seas, phytoplankton development and temperature have repeatedly been reported to structure bacterioplankton communities (Pinhassi et al., 2004; Rooney-Varga et al., 2005; Sapp et al., 2007; Rink et al., 2011). However, these two factors are not sufficient to explain the high diversity of marine bacterioplankton.

Reactive organic matter is released by phytoplankton and bacteria, often upon viral lysis and grazing, and is a major energy and carbon source for marine bacterioplankton communities. How structure and composition of bacterioplankton communities are influenced by spatial and temporal differences in organic matter (OM) composition is only poorly resolved. Numerous compounds in the vast pool of marine OM may support species-rich bacterioplankton communities, which are, in turn, able to efficiently remineralize large portions of the OM. In laboratory experiments, bacterioplankton exhibit specific OM uptake at a class and clade level (Pernthaler et al., 2002; Elifantz et al., 2005; Alonso-Sáez and Gasol, 2007). Single low molecular weight (LMW) compounds have been shown to structure bacterial communities (Gómez-Consarnau et al., 2012). The *in situ* structuring of bacterial communities is thought to be more complicated, involving a combination of available substrates, inorganic nutrients, physical factors and competition among bacteria (Gómez-Consarnau et al., 2012).

While large portions of marine OM are still chemically uncharacterized, carbohydrates have been identified as the largest fraction of characterized marine OM (Pakulski and Benner, 1994; Amon and Benner, 2003). Recent studies of the transcriptomic activity of marine bacterioplankton suggest that bacterial community composition and diversity are related to the genus-specific expression of metabolic genes (Teeling et al., 2012; Gifford et al., 2013), in particular those for carbohydrate-active enzymes. This allows for a succession of diverse bacterial strains that thrive on various forms of algal-derived OM during the spring phytoplankton bloom in the North Sea (Teeling et al., 2012).

Bacteria can take up LMW compounds as large as 0.6–0.8 kDa through porins and slightly larger compounds via TonB-dependent transporters (Weiss et al., 1991; Teeling et al., 2012) to meet their energy and carbon demands. Compounds with higher molecular weight must be hydrolyzed into smaller subunits by extracellular enzymes, prior to uptake. These are released by bacteria into the environment or attached to the outer cell membrane (Chróst, 1991). Heterotrophic bacteria have been found to favor HMW- over LMW-dissolved organic carbon (DOC), likely because HMW-DOC is less diagenetically altered and therefore more reactive and more usable (Amon and Benner, 1996). This underpins the importance of enzymatic hydrolysis as the first step in the bacterial degradation of complex carbohydrates. In addition to control through substrate availability, there is a close link between bacterioplankton species richness and diversity of β-glucosidase (β-Glcase) isoenzymes (Arrieta and Herndl, 2002), which likely influences the effectiveness of substrate utilization.

Understanding the interaction between bacterioplankton and diverse OM is important for our understanding of biogeochemistry (Azam et al., 1983). It is also an urgent matter, as there is growing evidence for the high potential of ocean acidification to alter phytoplankton OM production (Borchard and Engel, 2012; Engel et al., 2014), combined with changes in bacterial community composition (Allgaier et al., 2008; Krause et al., 2012; Sperling et al., 2013) and bacterial degradation activity (Grossart et al., 2006a; Tanaka et al., 2008; Piontek et al., 2013).

This field study aims to fill the gap between laboratory studies of single bacterial groups or carbohydrates and results inferred from *in situ* meta-transcriptomics. It investigates the influence of carbohydrate composition and concentration in seawater on the community structure of free-living (FL) and particle-associated (PA) bacteria by combining, for the first time, a detailed analysis of the composition of total combined carbohydrates (tCCHO; >1 kDa) using high-performance anion-exchange chromatography coupled with pulsed amperometric detection (HPAEC-PAD) and bacterial community analysis using Automated Ribosomal Intergenic Spacer Analysis (ARISA) and Catalyzed Reporter Deposition-Fluorescence *In Situ* Hybridization (CARD-FISH). Furthermore, it investigates the extracellular enzymatic activity of the bacterial community in relation to carbohydrates and the wide range of physicochemical and biological factors at the transition from winter to spring in the temperate North Sea.

## Materials and Methods

### Sampling

Surface water (∼1 m depth) was collected in a 10-L plastic carboy in the morning twice a week from February 2nd to May 18th 2010 from a research vessel at the long-term North Sea monitoring station “Helgoland Roads” between the islands of Helgoland (54° 11′03′′N, 7° 54′00′′E) in the German Bight, North Sea (see Wiltshire and Manly, 2004; Wiltshire et al., 2008).

### DNA Collection and Extraction

After pre-filtration through 10 μm-pore size filters, PA bacteria were collected by filtering 2 L of seawater through 3-μm filters (TCTP and TSTP, 47 mm, Millipore, Billerica, MA, USA). To collect FL bacteria, 500 mL of the resulting filtrate was filtered through a 0.2-μm filter (GTTP, 47 mm, Millipore, Billerica, MA, USA). All filters were cut into three equal pieces using sterilized pincers and scalpel and stored in a 2 mL-reaction tube at -20°C until processing.

The DNA from all three filter pieces was extracted simultaneously using lysozyme followed by phenol-chloroform-isoamylalcohol purification (as described in Sapp et al., 2007) with the addition of 0.002 mg of sterile Polyvinylpolypyrrolidone (PVPP) to bind larger contaminants. The DNA was re-diluted in 20 μL of PCR-grade water and the concentration measured using a NanoQuant Plate and an infinite M200 plate reader (both Tecan, Switzerland).

### Bacterial Community Analyses

Automated Ribosomal Intergenic Spacer Analysis (ARISA), as described in Sperling et al. (2013) was used to investigate PA (3–10 μm) and FL (0.2–3 μm) bacterial communities. In brief, the Intergenic Spacer (IGS)-region was amplified using the forward primer L-D-Bact-132-a-A-18 (5′-CCG GGT TTC CCC ATT CGG-3′) and the fluorescence-labeled reverse primer S-D-Bact-1522-b-S-20 (5′-TGC GGC TGG ATC CCC TCC TT-3′) described by Ranjard et al. (2000). Length separation was achieved in a 4300 DNA analyzer (Li-Cor, Bad Homburg, Germany) using polyacrylamide gels (5.5% ready to use matrix, Li-Cor Biosciences, Bad Homburg, Germany). The PCR products were amended with Blue Stop Solution (Li-Cor, Bad Homburg, Germany) in a 1:1 ratio, and the size standard IRDye^®^700, 50–1500 bp (Li-Cor, Bad Homburg, Germany), was applied. The wells were loaded with 0.25 or 0.5 μL of samples, depending on the amount needed to visualize bands clearly. Values obtained from the application of a 0.5 μL sample were divided by two prior to data analysis. The volume of applied sample was confirmed to have no significant effect on Sørensen-similarities in the PA- (ANOSIM, *R* = 0.12, *p* = 0.20) and FL- (ANOSIM, *R* = -0.15, *p* = 0.72) fractions. The software package BioNumerics (Applied Math, Sint-Martens-Latem, Belgium) was used to analyze the gel images. The bands with a length between 300 and 1500 bp were binned into size classes (termed “ARISA-band-classes”) to correct for minor length variations of the IGS regions between lineages with almost identical 16S rDNA-sequences. Bins of 3 bp were used for fragments up to 700 bp in length, bins of 5 bp were used for fragments between 700 and 1,000 bp, and bins of 10 bp were used for fragments larger than 1,000 bp (Brown et al., 2005; Kovacs et al., 2010). In this way, the total number of bands, i.e., community richness, was reduced by approximately 4.5 and 1% for the PA and FL bacterial data, respectively.

Values for Catalyzed Reporter Deposition Fluorescence *In Situ* Hybridization (CARD-FISH) of non-prefiltered samples on 0.2-μm filters were taken from Teeling et al. (2016).

### Analysis of Physicochemical and Phytoplankton Parameters

Physicochemical and phytoplankton parameters were investigated on a weekday basis (Monday–Friday) as part of the Helgoland Roads LTER time series, which is also accessible via the open database PANGEA^1^ (Wiltshire and Manly, 2004; Wiltshire et al., 2008). Surface water samples were investigated using standard colorimetric methods (Grasshoff et al., 1999). Salinity was measured by converting conduction, as measured with an inductive salinometer (GDTAutosal8400B Salinometer, Guildline, ON, Canada), to salinity using UNESCO tables (Cox and Culkin, 1976; Wiltshire et al., 2008). A fluorometer (bbe Moldaenke, Kiel-Kronshagen, Germany) was used to measure Chl *a* concentrations (Wiltshire et al., 2009). Inorganic nutrients (phosphate, silicate, ammonium, nitrate, and nitrite) were measured in the laboratory using the methods of Grasshoff et al. (1999) and Knefelkamp et al. (2007). Measurement of pH according to the National Bureau of Standards (NBS) scale was performed after samples were brought back to the laboratory. The pH difference due to the difference between *in situ* temperature and measurement temperature in the lab was corrected after the method of Gieskes (1969) using the following equation:

$${pH}_{in\,\;situ}={pH}_{measured}+0.0114\left(t_{measured}-t_{in\,\;situ}\right)$$

where *t*_measured_ is the temperature in °C, measured simultaneously with pH using a combined electrode (ProLab 3000 pH meter, IoLine pH combination electrode with temperature sensor) calibrated with standard buffer solutions (all materials: SI Analytics, Mainz, Germany).

### Particulate and Dissolved and Total Organic Carbon

Particulate carbon was collected on pre-combusted GF/F filters (25 mm, Whatman Nuclepore), which were stored at -20°C until analysis. The filtration volume (80–380 mL) was adjusted to the particle loading of the seawater. Filters were treated with 200 μL of carbon-free HCl (0.2 N) to remove inorganic carbon and dried at 60°C for approximately 48 h. After drying, filters were packed into small Zn-cartridges and analyzed using an elemental analyzer (Euro EA 3000, EuroVector Instruments & Software, Italy).

For analysis of DOC concentration, seawater was filtered through combusted (400°C, 4 h) GF/F filters (0.7 μm, Whatman), and 15 mL was sealed in combusted glass ampoules. Samples were stored at -80°C in the dark. Some of the ampoules were damaged during transport to the mainland, resulting in reduced temporal resolution. Acidified samples (pH 2, HCl) were analyzed using high-temperature catalytic oxidation (Sugimura and Suzuki, 1988) on a Shimadzu total organic carbon (TOC)-VCPH instrument with analytical precision better than 5% for three replicate samples. Accuracy was tested in each run against deep Atlantic seawater reference material (D.A. Hansell, University of Miami, FL, USA) and was better than 5%.

Total organic carbon (TOC) was calculated as the sum of particulate organic carbon (POC) and DOC.

### Carbohydrate Analysis

Analysis of total combined carbohydrates (tCCHO; >1 kDa), which are a portion of high molecular weight organic matter (HMW-OM), was performed according to Engel and Händel (2011). In brief, two replicate seawater samples (20 mL each) were transferred to pre-combusted glass vials using pre-rinsed disposable syringes and immediately stored at -20°C until processing of the samples. Samples were desalinated using membrane dialysis (1 kDa MWCO, Spectra Por). Carbohydrate monomers that were produced from acid hydrolysis (1 M HCl) and subsequent acid evaporation (N_2_), were analyzed on a Dionex ICS3000 system combining high performance anion exchange chromatography (HPAEC) and pulsed amperometric detection (PAD) using a Dionex CarboPac PA10 analytical column (2 × 250 mm) coupled to a Dionex CarboPac PA10 guard column (2 × 50 mm). The autosampler (Dionex AS50) was maintained at 8°C, and 17.5μL of sample was injected for each analysis. After every second sample, 17.5 μL of a mixed sugar solution was injected for standardization. Blanks (ultrapure water; Milli-Q) were analyzed using the same procedure used for the samples and subtracted from sample concentration. We identified 12 carbohydrate monomers (**Supplementary Table S1**). The neutral sugars arabinose (Ara), fucose (Fuc), galactose (Gal), glucose (Glc), and rhamnose (Rha) were investigated, as well as the co-eluting sugars mannose and xylose (Man/Xyl). The acidic sugars galacturonic acid (GalUA), gluconic acid (GlcA), glucuronic acid (GlcUA) and muramic acid (Mur) were measured. In addition, the concentrations of the two amino sugars galactosamine (GalN) and glucosamine (GlcN) were determined. The detection limit for the method was 1 nmol L^-1^. Values for all sugar components and standard deviations can be found in **Supplementary Table S2**.

### Extracellular Enzyme Activity

The activity of extracellular enzymes was determined by the use of fluorogenic substrate analogs (Hoppe, 1983). The rates of β-glucosidase, leucine (leu)-aminopeptidase, and alkaline phosphatase were assessed from the hydrolysis of 4-methylumbelliferyl-β-glucopyranoside, L-leucyl-4-methylcoum-arinylamid-hydrochlorid and 4-methylumbelliferyl-phosphate, respectively. The substrate analogs were added to seawater samples at final concentrations of 1, 5, 10, 20, 50, 80, 100, and 200 μmol L^-1^ to determine enzyme kinetics. To measure fluorescence, the infinite M200 plate reader (Tecan, Männedorf, Switzerland) was used. The fluorescence emitted by 4-methylumbelliferone (MUF) was detected at 365 nm excitation and 440 nm emission wavelengths, and that of 7-amino-4-methyl-coumarine (AMC) was detected at 380 nm excitation and 440 nm emission wavelengths. Fluorescence units were converted into concentrations of MUF or AMC after calibration with standard solutions. Enzymatic rates were calculated from the increase in MUF or AMC concentration, over time. An initial fluorescence measurement was conducted immediately after the addition of the substrate analog, followed by two measurements within 4 h of incubation in the dark at the approximate *in situ* temperature for the sampling day (1–8°C). The initial fluorescence was subtracted as background fluorescence from the fluorescence measured at each time point. The slope of the linear regression between incubation time and the concentration of the fluorescent marker was applied for rate calculations. Experimental data were fitted using the Michaelis–Menten equation to determine the maximum velocity (*V*_max_) of the enzymatic reactions. To estimate the amount of substrate hydrolyzed by β-glucosidase (β-Glcase) during the post-bloom phase, we calculated the enzymatic rate for each day using a mean post-bloom concentration of 2.13 μmol L^-1^ Glc. The resulting mean rate (83.05 nmol L^-1^ d^-1^) was multiplied by the number of days (26) in the post-bloom phase.

### Statistical Analysis

For multivariate statistical analyses, the software package PRIMER v.6 and the add-on PERMANOVA+ (both PRIMER-E, Auckland, New Zealand) were used. The analyses were performed using Sørensen-similarity-matrices generated from square-root transformed ARISA-band-class data. To test for community assemblage differences between phytoplankton bloom phases, permutational multivariate analysis of variance (PERMANOVA) was applied. Distance-based multivariate multiple regression (DistLM) was used to calculate correlations of community composition to environmental factors, and distance-based redundancy analysis (dbRDA) was used to visualize these correlations. For this analysis, the highly collinear combined carbohydrates (colCHO, Pearson *r* > 0.8) Fuc, Rha, Gal, Glc, GalN, GlcN, and GlcUA were pooled.

## Results

### Phytoplankton Bloom Development

**FIGURE 1:**
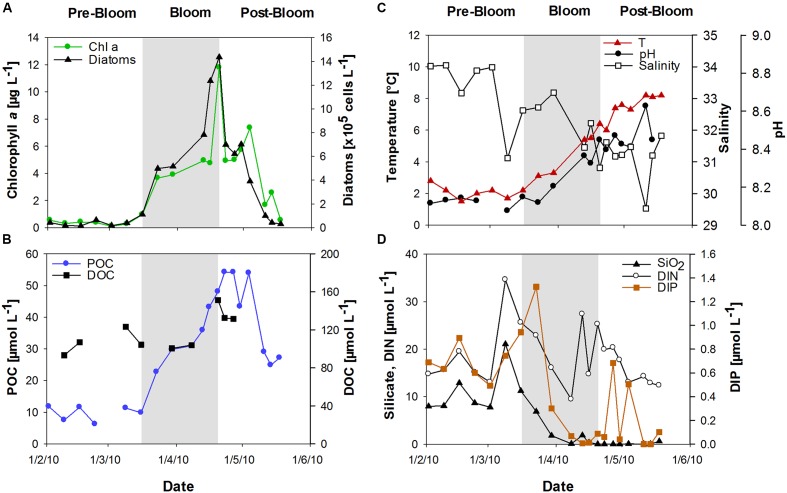
**Development of the spring bloom at Helgoland Roads in 2010. (A)** Chlorophyll *a* (Chl *a*) concentrations and diatom abundance, **(B)** Temperature, salinity and seawater pH, **(C)** Particulate organic carbon (POC) and dissolved organic carbon (DOC), **(D)** Inorganic nutrients comprising silicate (SiO_2_), dissolved inorganic nitrogen (DIN) and dissolved inorganic phosphorous (DIP).

Chlorophyll *a* (Chl *a*) was used to determine the state of the phytoplankton bloom. After low Chl *a* concentrations (<1 μg L^-1^) in February, a diatom-dominated spring phytoplankton bloom developed at the long-term monitoring station “Helgoland Roads” (54° 11′03′′N, 7° 54′00′′E). Bloom development started in mid-March and reached Chl *a* values of up to 12 μg L^-1^ in April (**Figure 1A**). The onset of the bloom coincided with a strong peak in dissolved inorganic phosphate (DIP) concentration and a slight increase in sea surface temperature (**Figures 1C,D**). The bloom development was accompanied by rising seawater pH. Salinity decreased from approximately 34 in February to approximately 31 at the end of April (**Figure 1B**). The phytoplankton bloom terminated in late April, likely due to the depletion of DIP and silicate (SiO_2_) (**Figure 1D**).

### Organic Carbon and Carbohydrate Composition

**FIGURE 2:**
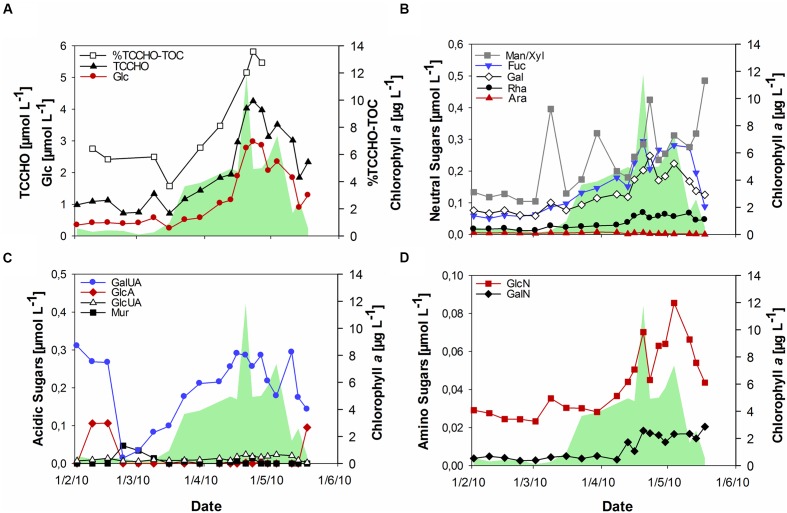
**Concentrations of combined carbohydrates >1 kDa (TCCHO) during the spring bloom. (A)** Concentrations of TCCHO and combined glucose (Glc) and share of TCCHO in TOC (% TCCHO-TOC, TOC = POC + DOC) **(B)** Concentrations of neutral sugars including mannose/xylose (Man/Xyl), fucose (Fuc), galactose (Gal), rhamnose (Rha), and arabinose (Ara) **(C)** Concentrations of acidic sugars including gluconic acid (GlcA), glucuronic acid (GlcUA), galacturonic acid (GalUA), and muramic acid (Mur) **(D)** Amino sugars including glucosamine (GlcN) and galactosamine (GalN). The green shaded area represents concentrations of chlorophyll *a*.

During the bloom, POC increased with diatom abundance, reaching a maximum of 54 μmol L^-1^ (**Figure 1B**), while DOC showed a maximum value of 151 μmol L^-1^ at the peak of the bloom. TOC values were calculated as the sum of POC and DOC and reached maximum values of 135 and 187 μmol L^-1^ before and after the phytoplankton bloom, respectively. Bloom development resulted in the accumulation of tCCHO, which contributed up to 14% of TOC in the post-bloom phase (**Figure 2A**). The subsequent decline in tCCHO and POC lagged behind the decrease in diatom cell numbers and Chl *a* (**Figures 1A,B** and **2A**). The carbon contribution of tCCHO to TOC strongly increased during the bloom (**Figure 2A**). Several carbohydrates in HMW-OM showed very similar temporal dynamics (Pearson *r* > 0.8) during spring 2010. Therefore, for the statistical analysis, glucose, galactose, fucose, rhamnose, galactosamine, glucosamine, and glucuronic acid were pooled into a single factor and designated as collinear CHO (colCHO). Its concentration strongly increased toward the end of the phytoplankton boom and maintained high values during the early post-bloom phase (**Figures 2B–D**). In contrast, Ara showed concentrations near the detection limit during the entire study, while GalUA revealed strong dynamics unrelated to the phytoplankton bloom. Throughout the bloom, the composition of tCCHO was dominated by neutral sugars (60–90 mol%). Glc alone contributed an average 53 mol%, with maximum concentrations of 3 μmol monomer equivalent L^-1^ in the late bloom and post-bloom phase (**Figure 2A**). Concentrations of Man/Xyl, Fuc and Gal increased with the onset of the phytoplankton bloom and maintained high values during the post-bloom phase, though maximum concentrations of 0.2–0.4 μmol L^-1^ were approximately an order of magnitude lower than that of Glc (**Figure 2B**). Man/Xyl and Fuc showed similar mean concentrations of 0.13 and 0.16 μmol monomer equivalent L^-1^, respectively. In the late post-bloom period, Man/Xyl strongly increased, while Fuc decreased rapidly. Rha and Ara showed comparatively low concentrations ≤ 0.1 μmol L^-1^ throughout the investigation period.

The acidic sugar GalUA was a major component of tCCHO before the onset of the bloom in February, followed by strongly declining concentrations in March (**Figure 2C**). Concentrations increased again until the end of the phytoplankton bloom, reaching values of 0.2–0.3 μmol L^-1^, comparable to the concentrations of most neutral sugars, in April (**Figure 2C**). The other acidic sugars GlcA, Mur, and GlcUA were only present in very low concentrations (below 0.02 μmol L^-1^). Of those, only GlcUA increased slightly in concentration toward the end of the bloom and maintained an elevated level during the post-bloom. Amino sugars were only present at low concentrations (below 0.1 μmol L^-1^) (**Figure 2D**). However, both, GalN and GlcN concentrations increased toward the end of the bloom, with GlcN reaching values of up to 0.07–0.08 μmol L^-1^, higher than the concentrations of most acidic sugars during the late bloom and post-bloom phases.

### Bacterial Community Composition

**FIGURE 3:**
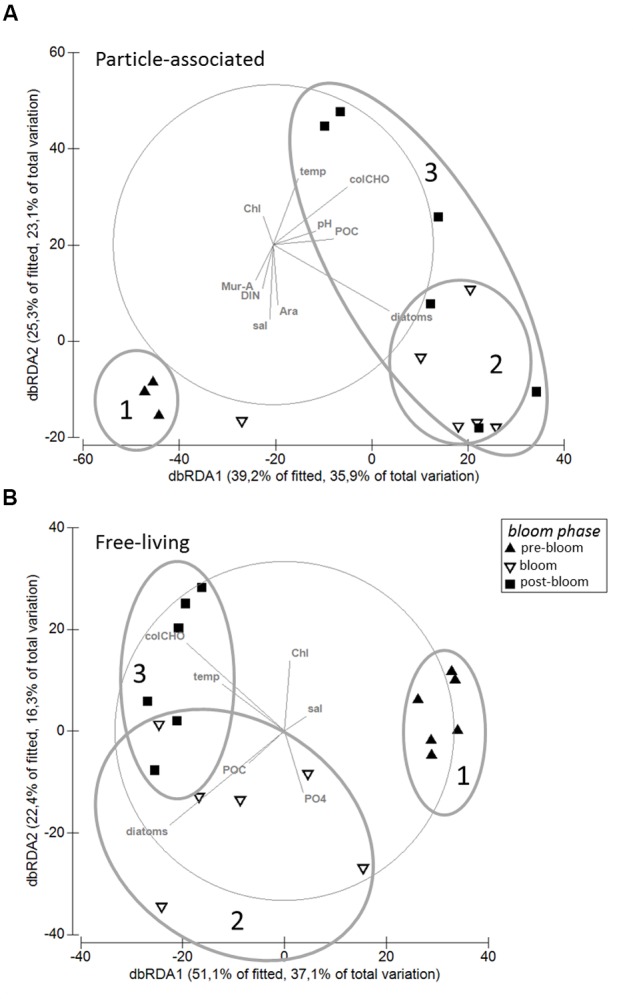
**Distance-based Redundancy Analysis (dbRDA) of the relationship between environmental parameters and (A)** particle-associated and **(B)** free-living bacterial communities. The analysis is based on Sørensen-similarity-matrices of square root-transformed data. Ovals (1–3) illustrate community dynamics during the different phytoplankton bloom phases.

**FIGURE 4:**
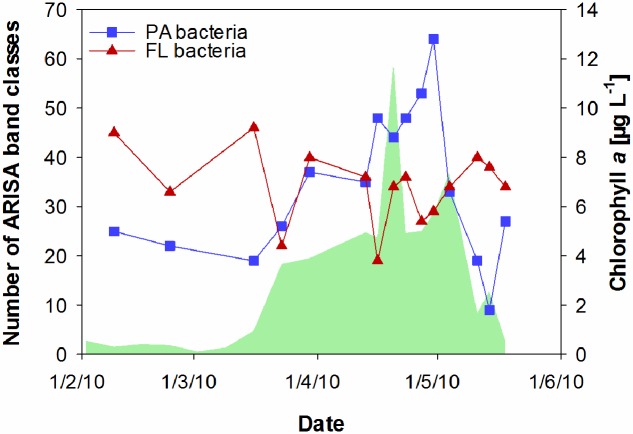
**Temporal changes in the richness of particle-associated (PA) bacteria and free-living (FL) bacteria at Helgoland Roads, derived from ARISA band classes.** The green shaded area represents concentrations of chlorophyll *a*.

Particle-associated and FL bacterial communities were significantly different during the post-bloom phase (PERMANOVA; *p* = 0.008, 400 permutations) but were similar during the pre-bloom (PERMANOVA; *p* = 0.092, 10 permutations) and bloom phases (PERMANOVA; *p* = 0.141, 414 permutations). Both, PA and FL bacterial community significantly changed between phytoplankton bloom phases (PERMANOVA; *p* = 0.003, 992 permutations and *p* < 0.001; 999 permutations, respectively), revealing distinct temporal successions. DistLM shows that the development of PA communities is significantly related to three environmental variables. Apart from an abrupt change along a POC gradient (explaining ∼31% of total community variability) at the onset of the phytoplankton bloom, PA bacteria showed the strongest community development during the post-bloom phase, following a gradient of temperature (explaining ∼20%) and carbohydrates (colCHO, explaining ∼9%) (**Table 1**, **Figure 3**). The FL bacterial community composition strongly changed during the ongoing phytoplankton bloom and during the post-bloom phase, mainly tracking with diatom abundance (∼14%) and temperature (∼34%), respectively (**Table 1**, **Figure 3**). In total, significant environmental factors explained ∼60 and ∼48% of the variability in PA and FL communities, respectively. The richness of PA bacteria increased with rising Chl *a* and tCCHO concentrations, revealing a linear correlation (*R*^2^ = 0.3779, *p* = 0.0093) between colCHO concentration and PA bacterial richness (**Supplementary Figure S1**). The PA richness reached a maximum approximately 1.5 weeks after the breakdown of the bloom at persistently high concentrations of tCCHO and was followed by a steep decrease in both richness and tCCHO thereafter (**Figures 2A** and **4**). In contrast, the richness of FL bacteria did not resemble the temporal development of Chl *a* or diatom cell numbers during the bloom (**Figure 4**).

**Table 1:** DistLM showing the correlation of ARISA-band-diversity (Sørensen) to environmental factors (sequential addition to the model).

Variable	Adj. *R*^2^	Pseudo-*F*	*P*	Prop. %	Cumul. %
**Particle-associated**				
POC	0.26115	59.48	**0.001**	31.39	31.39
Temp	0.42877	48.15	**0.006**	19.65	51.04
colCHO	0.49407	25.49	**0.009**	9.21	60.25
pH	0.52738	17.76	0.075	5.99	66.24
Diatoms	0.55793	1.69	0.104	5.34	71.58
Sal	0.58148	15.06	0.169	4.50	76.08
DIN	0.60484	1.47	0.200	4.16	80.24
Ara	0.63418	15.61	0.194	4.08	84.32
Mur-A	0.66667	1.59	0.213	3.77	88.10
Chl	0.70459	16.42	0.221	3.46	91.56
**Free-living**					
Temp	0.29753	82.00	**0.001**	33.89	33.89
Diatoms	0.40907	40.20	**0.001**	13.98	47.86
PO4	0.42655	14.57	0.181	4.92	52.78
Chl	0.46659	2.05	0.073	6.44	59.21
Sal	0.47932	13.18	0.27	4.04	63.25
colCHO	0.5011	15.24	0.175	4.47	67.72
POC	0.53377	17.71	0.13	4.86	72.58

*Significant variables (*p* < 0.05) are given in bold*.

Of the investigated microbial clades (CARD-FISH), only GAM42 (*Gammaproteobacteria*) showed a direct positive correlation to colCHO and to GalUA (**Supplementary Table S3**). Notably, these also correlated to temperature and pH, as well as POC and Chl *a*. Furthermore, they were correlated positively to all investigated extracellular enzymes. Although in total, *Roseobacter* did not correlate to any investigated parameter, the SAR11 subgroup correlated to pH and silicate. Bacteroidetes correlated to Chl *a*.

### Extracellular Enzyme Activity

**FIGURE 5:**
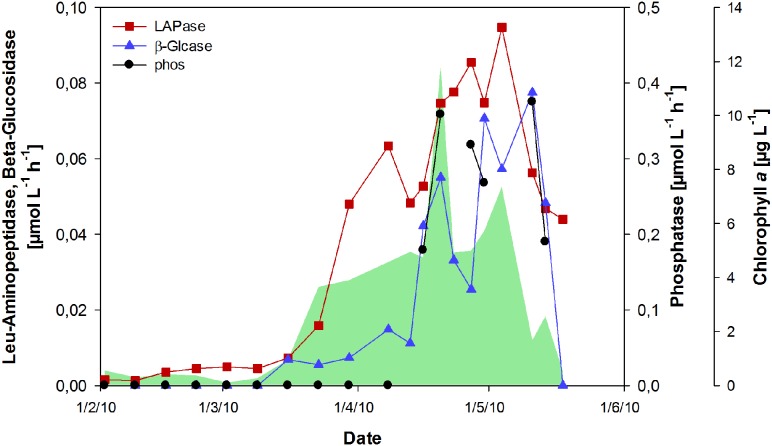
**Activity of hydrolytic extracellular enzymes during the spring bloom.** Rates for leucine (Leu)-aminopeptidase (LAPase), β-glucosidase (β-Glcase), and phosphatase (phos) are shown. The green shaded area represents concentrations of chlorophyll *a*.

The activity of extracellular enzymes was near the detection limit from February to mid-March (**Figure 5**). With the onset of the phytoplankton bloom, leucine-aminopeptidase (LAPase) activity increased and maintained high levels during the bloom, reaching a maximum of 0.95 μmol L^-1^ h^-1^ in the post-bloom phase. The activity of β-glucosidase (β-Glcase) increased toward the end of the bloom, concurrent with a strong increase in tCCHO concentrations (**Figure 2A**). A maximum rate of 0.08 μmol L^-1^ h^-1^ was determined on May 11th. The amount of substrate hydrolyzed by β-Glcase during the post-bloom phase was estimated as approximately 2.16 μmol L^-1^, equivalent to approximately 72% of the total combined glucose that accumulated during the bloom (**Figure 2A**). The rates of extracellular phosphatase (phos), increased rapidly after phosphate depletion (**Figures 1D** and **5**) at the end of the bloom, indicating the enhanced use of organic phosphorous by bacteria and phosphatase-releasing phytoplankton groups. The co-incidence of phosphate depletion with a pronounced increase in phosphatase activity correlates well with the increase in phosphate transporters, as reported in Teeling et al. (2012) for the year 2009. This implies that the enzymatic degradation of organic phosphorous was repressed by the accumulation of its end product.

All enzyme activities were positively correlated with seawater pH, temperature and POC but were negatively correlated to nutrients (**Table 2**). Phos was positively correlated to DOC. In general, all neutral tCCHO except Ara and Man/Xyl showed strong (*r*_s_ > 0.7) to very strong (*r*_s_ > 0.9) positive correlations with LAPase, Glcase, and phos. Furthermore, amino sugars correlated well with enzyme activities. β-Glcase and LAPase activity were very strongly (*r*_s_ > 0.9) correlated with Fuc, while LAPase was correlated very strongly (*r*_s_ > 0.9) with Gal. Man/Xyl correlated with only amino-peptidase and phos, but not β-Glcase. The amino-sugars correlated well with enzyme activities. Glc-URA was the only acidic sugar showing a significant correlation with enzyme activities.

**Table 2:** Spearman correlation (*p* < 0.05) of different carbohydrates and environmental factors to extracellular enzyme activity of β-glucosidase (β-Glcase), leucine amino peptidase (LAPase), and phosphatase (phos).

	β-Glcase	LAPase	phos
**Carbohydrates**			
Ara	ø	ø	-
Fuc	+++	+++	++
Gal	++	+++	++
Glc	++	++	++
Man/Xyl	ø	+	+
Rha	++	++	++
GalAM	+	+	++
GlcAM	++	++	++
GlcA	ø	ø	ø
MurA	ø	ø	ø
GalURA	ø	ø	ø
GlcURA	+	+	+
**Environmental parameters**			
pH	++	++	++
SECCI	-	ø	ø
Temp	++	++	++
Sal	-	-	--
SiO_2_	--	--	--
PO_4_	-	-	-
NO_2_	--	--	--
Diatoms	+	++	ø
Chl *a*	++	++	+
DOC	ø	ø	++
POC	+++	+++	++

*Ø = no significant correlation; + = modest positive correlation (*r*_s_ > 0.4), ++ = strong positive correlation (*r*_s_ > 0.7), +++ = very strong positive correlation (*r*_s_ > 0.9), - = modest negative correlation (*r*_s_ < -0.4), -- = strong negative correlation (*r*_s_ < -0.7)*.

## Discussion

The present study is the first field study combining a detailed analysis of the monomeric composition of polysaccharides in seawater with an investigation of bacterial community composition and the activity of hydrolytic extracellular enzymes. Multivariate statistics were applied to investigate the relationship between the temporal development of the bacterioplankton community and environmental parameters during the spring bloom. Correlations cannot prove or disprove functional relationships but are a valuable measure of the relevance of certain environmental controls on communities and activities. The discussion addresses the temporal development of carbohydrate composition, bacterioplankton community composition and enzyme activities. Furthermore, it aims to reveal potential links between the succession of bacterioplankton and major abiotic and biotic factors during the spring bloom at Helgoland Roads.

### The Contribution of Combined Carbohydrates to Organic Matter during Phytoplankton Bloom Development

Phytoplankton spring blooms are events of high OM input into marine systems. The diatom spring bloom investigated at Helgoland Roads in 2010 showed a maximum Chl *a* concentration of approximately 12 μg L^-1^, similar to concentrations reported for the southern North Sea (Veldhuis et al., 1986) but twofold lower than at Helgoland Roads in 2009 (Teeling et al., 2012). The investigated bloom sustained an average POC concentration of 29 μmol C L^-1^, well within the range observed previously in the German Bight (Rink et al., 2011) and comparable to values found in springtime in the eastern North Atlantic (Engel et al., 2012). Average DOC concentrations of 116 μmol C L^-1^ and tCCHO concentrations of 2 μmol monomer equivalent L^-1^ determined in our study are approximately 30% higher than in the eastern North Atlantic Ocean (Engel et al., 2012). The contribution of tCCHO to TOC at Helgoland Roads averaged 8.3%, similar to the percentage of neutral sugars in the TOC of the NW-Mediterranean Sea in April (Jones et al., 2013) but approximately fourfold higher than at the oligotrophic Bermuda Atlantic Time-Series station (BATS) in June (Kaiser and Benner, 2009). The results of this study reveal collinear temporal dynamics of most monomeric components in combined carbohydrates (colCHO) during the growth and decline of the diatom spring bloom at Helgoland Roads. The carbohydrate composition was dominated by high shares of combined glucose. The molar percentages of the individual neutral sugars in tCCHO are more similar to the carbohydrate composition in Oregon inshore waters and Arctic waters than to the carbohydrate composition in the equatorial Pacific Ocean or the Sargasso Sea (Borch and Kirchman, 1997; Rich et al., 1997; Kirchman et al., 2001).

The contribution of tCCHO to TOC increased strongly during the late bloom phase. The coincidence of increasing tCCHO concentrations and the depletion of dissolved inorganic phosphorous and dissolved silicate suggests that enhanced phytoplankton exudation substantially contributed to the production of carbohydrate-rich OM. Laboratory studies have shown that large diatom species, in particular, release high amounts of carbohydrate-rich exudates when grown under nutrient-limited conditions (Engel et al., 2002; Passow, 2002). Diatom exudates are important precursors for the formation of marine particles. Exuded polysaccharidic gels, like transparent exopolymeric particles (TEP), provide surfaces for bacterial colonization and subsequent degradation. Furthermore, TEP play a decisive role in the formation of macroscopic aggregates that promote flocculation and sinking of biogenic material at the end of bloom events (Passow et al., 1994). Acidic sugars represent a substantial fraction of carbohydrates in TEP (Alldredge et al., 1993; Mopper et al., 1995; Passow, 2002). GalUA and GlcUA, the two acidic sugars analyzed in this study, showed very different temporal developments. While concentrations of GlcUA increased only slightly in the post-bloom phase, strong increases in GalUA coincided with rising Chl *a* concentrations. This accumulation of GalUA during the bloom supports the assumption of a high proportion of exudates derived from phytoplankton at Helgoland Roads. In addition to exudation, grazing has potentially contributed to the release of dissolved carbohydrates. The relatively low Chl *a* concentrations recorded in our study indicate a top-down control of phytoplankton by zooplankton (Lohmann and Wiltshire, 2012) that tends to lead to a release of complex carbohydrates from sloppy feeding (Storm et al., 1997).

The complex DOM released from phytoplankton is highly strain specific (Becker et al., 2014) and likely explains most of the associations of specific plankton bacteria with particular phytoplankton strains (e.g., Hahnke et al., 2013). Phytoplankton-derived polysaccharides have been shown to support clade-specific proliferation of bacterioplankton (Taylor et al., 2014). However, the species richness of heterotrophic bacteria is higher than that of autotrophs, suggesting a complex but not strictly species-related interaction between phytoplankton-derived OM and heterotrophic bacterioplankton. This is supported by the finding that relatively few strains of heterotrophic bacteria are associated with distinct phytoplankton communities (Teeling et al., 2016) and that most OM is used by generalists (Rink et al., 2007).

### Succession of Bacterioplankton

A strong increase in almost all sugars in tCCHO toward the end of the bloom suggests an increase in ecological niches available to heterotrophic bacteria (Alonso-Sáez and Gasol, 2007; Teeling et al., 2012; Gifford et al., 2013). Indeed, a rapid increase in the richness of the PA bacterial community (approximately 50%) was observed within the 14 days after diatom cell numbers peaked. In that period, the concentration of colCHO (the combined concentration of collinear sugars) was a significant factor impacting the succession of the PA bacterial community. Our results suggest that the influence of carbohydrate concentration on bacterial growth and activity observed earlier (e.g., Borch and Kirchman, 1997; Rich et al., 1997; Piontek et al., 2011) might be induced by not only increasing metabolic rates at the cellular level but also changes in bacterial communities toward efficient carbohydrate degradation. A previous study conducted during the spring bloom at Helgoland Roads showed the taxonomically distinct expression of TonB-dependent transporters, implying the specialization of populations for the successional degradation of carbohydrates in different size ranges (Teeling et al., 2012). Recruitment of FL bacteria to particles cannot explain the rise in PA richness during the post-bloom phase, as PA and FL communities were very similar throughout the phytoplankton bloom. Hence, organic particles rich in carbohydrates provide beneficial niches for a distinct and diverse PA bacterial community that sustains high rates of hydrolytic enzyme activity. Furthermore, approximately three times more new ARISA band classes were detected during the post-bloom phase than tCCHO components rise in concentration. This suggests that only part of the rise in richness could be explained by specialists growing on specific monomeric carbohydrates. It seems likely that several bacterial taxa thrive on the same component or use a specific combination of different components.

Our CARD-FISH analysis, which did not distinguish between PA and FL fractions, revealed that *Gammaproteobacteria* (GAM42) were positively correlated to colCHO and showed higher abundances toward the end of the bloom. Further correlations of *Gammaproteobacteria* with POC and Chl *a* underline the importance of fresh OM derived from phytoplankton production for the growth of this group, which is a prominent member of bacterial communities associated with marine particles (Bižić-Ionescu et al., 2014). For the first time, this study shows that the abundance of *Gammaproteobacteria* is not only related to bulk OM but also to a specific OM component. TCCHO are considered a labile to semi-labile source of organic carbon, with turnover times ranging from days to months depending on molecular weight and structure. GAM42 is also correlated positively to β-Glcase, LAPase, and Phos in our study, indicating an active role in the enzymatic hydrolysis of organic particles and thereby in the transfer of carbon from the particulate to the dissolved pool.

Changes in community composition related to the concentrations of combined carbohydrates and POC during the post-bloom phase strongly suggest a partially substrate-controlled succession. Overall, the investigated factors in our study explain approximately 60 and 48% of the community variability in PA and FL bacterioplankton, respectively. Interestingly, the carbohydrate composition determined in this study does not explain the succession of bacterial communities, inferred from ARISA and CARD-FISH analysis, during the period of strongly rising Chl *a* concentrations (March 16th–April 20th, 2010). In addition, approximately 75% of PA ARISA band classes did not correlate with the development of carbohydrate concentrations in any bloom phase. This might be explained by the fact that carbohydrates analyzed in this study are >1 kDa and thus part of the HMW-OM. The most labile fraction of freshly produced OM includes LMW compounds, which were shown to strongly affect bacterial community composition in *in vitro* experiments (Gómez-Consarnau et al., 2012). It can be suggested that freshly produced LMW-OM, which is not included in our analysis, had a stronger influence on bacterial community structure during earlier bloom phases before HMW-OM began to accumulate. In addition to the factors POC, colCHO and diatom abundance (related to phytoplankton productivity), temperature had a significant effect on the composition of bacterioplankton, indicating a multifactorial environmental control of bacterioplankton by seasonal changes. In line with previous findings, FL bacteria, which are more directly exposed to conditions in the water body, were found to be susceptible to temperature changes during spring bloom development (Sapp et al., 2007). Accordingly, the FL bacterial community richness is less dynamic than the PA community, and no correlation to the composition or concentration of tCCHO was observed. Furthermore, the hydrographic history of the water mass should be considered as an important factor in shaping bacterioplankton communities. The abundance of SAR11, which constituted up to 40% of total bacterial cells in this study, is related to salinity changes in the southern North Sea that track with the dynamics of water masses (Sperling et al., 2012). Hence, the origin and trajectories of water masses can affect temporal developments observed in time series studies, if complex current patterns, such as those in the shallow southern North Sea prevail.

### Activity of Hydrolytic Extracellular Enzymes

Extracellular enzyme activities were dependent on the phyto plankton bloom phase and were correlated to Chl *a*, POC, pH and temperature. Enzymatic rates show positive correlations to the fluorescence intensity of many PA ARISA band classes that can serve as a semi-quantitative measure of the abundances of individual bands (**Supplementary Figure S2**). In addition, the high activity of extracellular β-Glcase was observed well after the Chl *a* and tCCHO peaks, when PA bacterial richness was high. This indicates that for β-Glcase, high PA bacterial richness facilitates the enzymatic turnover of tCCHO. It is likely that the richer PA bacterial community produces not only high amounts of β-Glcase but also a high diversity of β-Glcase iso-enzymes (Arrieta and Herndl, 2002), thereby further enhancing the efficiency of carbohydrate hydrolysis. A study analyzing annotated prokaryotic genomes revealed that the capacity to produce extracellular enzymes varies at fine-scale phylogenetic resolution (Zimmerman et al., 2013). In line with previous studies, it can be suggested that PA bacteria are the main producers of extracellular enzymes, as they are closer to their substrates or even reside in semi-enclosed environments where they profit from the effort of producing extracellular enzymes. In contrast, FL bacteria do not appear to invest as much in extracellular enzymes, but preferentially consume carbohydrates that are suitable for direct uptake and, in some cases, subsequent hydrolysis (Teeling et al., 2016).

The rates of β-Glcase determined in the post-bloom phase are sufficiently high to drive the observed loss of tCCHO during this late bloom stage. In late April, a maximum tCCHO amount of approximately 4 μmol L^-1^ accumulated and was subsequently reduced by 2 μmol L^-1^ during the post-bloom phase. This loss of tCCHO matches well with β-Glcase activity integrated over the post-bloom phase, which could sustain the hydrolytic release of 2.16 μmol Glc L^-1^ within the 26 days of the post-bloom phase. In accordance with an earlier study (Arrieta and Herndl, 2002), high rates of extracellular β-Glcase were maintained even after the decline in bacterial richness. This can be explained by prolonged half-lives for excreted enzymes, in the range of days–weeks (Steen and Arnosti, 2011). The decay of the phytoplankton bloom did not result in increased FL bacterial richness, and only a few correlations of FL ARISA band classes to extracellular enzymes were found. This is in accordance with an earlier study that found no increased abundance of carbohydrate-active enzymes during post-phytoplankton spring blooms in FL bacteria at the same sampling site (Teeling et al., 2016). Therefore, it can be suggested that PA and FL bacteria mainly thrive on different forms of carbohydrates, and FL bacteria potentially profit from the pre-hydrolyzed substrates released from particles.

Little is known about the bioavailability of specific carbo hydrates in seawater for heterotrophic marine bacterioplankton *in situ*. In our study, high concentrations of Glc, Fuc and Gal were associated with high PA bacterial diversity. Concentrations of these sugars in tCCHO clearly decreased during the post-bloom phase, suggesting preferential degradation via bacterial activity. This observation partly corroborates findings from the Bay of Biscay, which also indicated Fuc and Gal to be actively consumed CHO. In contrast, Glc appears to be very actively processed during our study, but less dynamic in the Bay of Biscay (Engel et al., 2012). The availability of monomeric and oligomeric sugars for bacterioplankton consumption is, to a large extent, dependent on the degradability of preceding polymers. A *Bacteroidetes* isolate from North Sea surface waters, *Gramella forsetii* KT0803, showed polymer-specific transcription of polysaccharide utilization loci (PULs) for laminarin and alginate that comprised genes of surface-exposed proteins such as oligomer transporters, substrate-binding proteins, and carbohydrate-active enzymes (Kabisch et al., 2014). In particular, laminarin is a widespread polymer in the ocean that is found as a storage glucan in diatoms and brown algae, among others. It consists of glucose monomers linked by β-glycosidic bonds that can be hydrolyzed by β-Glcase. Its homopolysaccharidic structure (i.e., consisting of only one type of monomer) further facilitates efficient hydrolysis by exo-enzymes. Homopolysaccharides have been found to be preferentially utilized over heteropolysaccharides (Amon and Benner, 2003). Heteropolysaccharides consisting of Rha, Fuc, Xyl, Man, and Gal are mainly part of the cell wall (Alderkamp et al., 2007) and are excreted as extracellular polymeric substances (Hama and Yanagi, 2001). Both glucanes and heteropolysaccharides appear to contribute to the rise in carbohydrates observed during the bloom in our study. Apart from Glc in glucanes, Fuc and Gal appear to be part of labile polymers. In addition to the monomeric composition of polysaccharides, also the molecule structure of the substrate has been shown to co-determine the accessibility to extracellular enzymes (Pantoja and Lee, 1999; Arnosti, 2000, 2004). Laminarinase enzymes, for example, showed minimal activity on substrates, with similar glucosidic bonds to those of laminarin, but different sizes and secondary and/or tertiary structures, revealing that the hydrolysis rates among substrates of similar sizes but differing structures can vary considerably (Alderkamp et al., 2006). Overall, it can be assumed that the metabolic capacities of the bacterial community adapted to the chemical properties of available polysaccharides, resulting in efficient polysaccharide degradation during the spring bloom at Helgoland Roads.

## Conclusion

The present study shows a correlation between the concentration of combined carbohydrates in HMW-OM and the diversity of PA bacteria during the development of a spring bloom, suggesting that the availability of carbohydrates contributes to the multifactorial control of marine bacterioplankton communities. Our study is in line with earlier findings demonstrating the importance of PA bacteria for total community activity (Alldredge and Silver, 1988; Herndl, 1988; Grossart et al., 2006b; Lyons and Dobbs, 2012). This highlights the additional value of analyzing bacterioplankton community composition in size-fractionated samples. As inferred from decreasing concentrations during the post-bloom phase, glucose, fucose, and galactose were preferentially utilized sugars in HMW-OM. There is growing evidence that phytoplankton primary production and OM release is susceptible to ocean change. Climate models project the shoaling of upper mixed layer depth as a consequence of sea-surface warming. Changes in the mixed layer depth can affect primary production and the timing of spring blooms and thus the pool of OM that is subject to bacterial remineralization. A mesocosm study further showed that phytoplankton growth under increasing temperature accelerates carbohydrate accumulation (Engel et al., 2011). In addition to warming, the dissolution of increasing anthropogenic CO_2_ in seawater, referred to as ocean acidification, can significantly affect phytoplankton productivity. A mesocosm experiment revealed that elevated *p*CO_2_ increased primary production in Arctic plankton communities (Engel et al., 2013). In the same experiment, the PA bacterial community richness was shown to be higher in mesocosms at elevated *p*CO_2_ (Sperling et al., 2013). Increasing exudation has been suggested as a physiological strategy for *Emiliania huxleyi*, a bloom-forming coccolithophore, to grow under a condition of elevated *p*CO_2_ and low nutrient availability (Borchard and Engel, 2012). Combining results from fieldwork and experiments, it can be suggested that carbohydrates, the primary product of photosynthesis, have high potential to mediate the effects of ocean change on bacterioplankton community structure and function (**Supplementary Figure S3**).

## Author Contributions

MS developed the study concept and conducted the sampling as well as most of the sample- and data analysis. He also wrote the manuscript. JP helped develop the study concept, provided methodological support, especially with acquisition and analysis of extracellular enzyme data, and contributed to the manuscript. AE and AW provided lab space and financial and methodological support, helped develop the study concept and contributed to the manuscript. KW provided phytoplankton- and physicochemical data and contributed to the manuscript. JN provided data on dissolved organic carbon and contributed to the manuscript. GG provided lab space and financial and methodological support, helped develop the study concept, supported the statistical analysis and contributed to the manuscript.

## Conflict of Interest Statement

The authors declare that the research was conducted in the absence of any commercial or financial relationships that could be construed as a potential conflict of interest.

## Footnotes

1 http://www.pangea.de

## Supplementary Material

FIGURE S1 **Richness of particle-associated (3–10 μm) bacterioplankton vs. the concentration of colCHO (*R*^2^ = 0.3779, *p* = 0.0093)**.

FIGURE S2 **Percent of particle-associated and free-living ARISA band classes (fluorescence intensity) correlating (Spearman Rank Correlation; *r*_s_ > 0.5, *p* < 0.05) to the maximum velocity (*V*_max_) of the extracellular enzymes β-glucosidase (β-Glcase), leucine aminopeptidase (LAPase) and phosphatase (phos) during spring 2010 at the Helgoland Roads sampling station**.

FIGURE S3 **Schematic representation of a mechanism for secondary effects of ocean change on bacterioplankton community and activity**.

TABLE S1 **Analyzed carbohydrates and abbreviations**.

TABLE S2 **Concentrations of tCCHO components in nmol L^-1^**.

TABLE S3 **Spearman correlation (*p* < 0.05) of FISH-counts to environmental factors (abiotic and biotic), concentrations of selected carbohydrates, and extracellular enzyme activities of β-glucosidase (β-Glcase), leucine amino peptidase (LAPases) and phosphatase (phos)**. Ø = no significant correlation; + = modest positive correlation (*r*_s_ > 0.4), ++ = strong positive correlation (*r*_s_ > 0.7), +++ = very strong positive correlation (*r*_s_ > 0.9), - = modest negative correlation (*r*_s_ < -0.4), -- = strong negative correlation (*r*_s_ < -0.7).
