# Supplementary figures and images for: Combined Carbohydrates Support Rich Communities of Particle-Associated Marine Bacterioplankton
Source: Front Microbiol. 2017 Jan 31;8:65. doi: 10.3389/fmicb.2017.00065 (PMC5281597; doi:10.3389/fmicb.2017.00065)

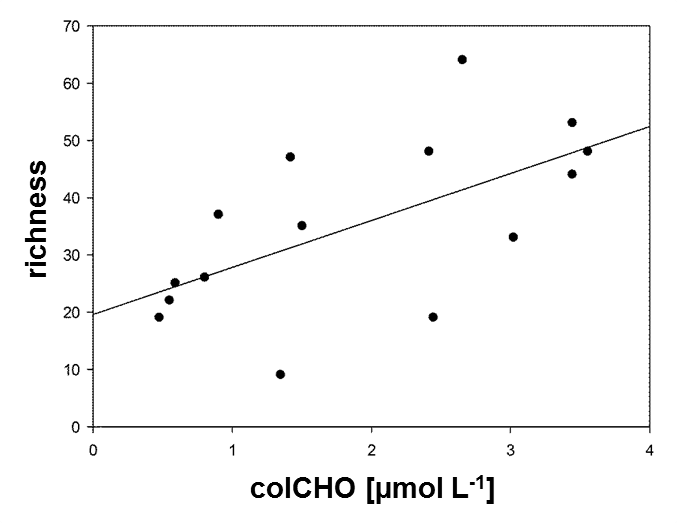

Supplement: FIGURE S1 — Richness of particle-associated (3–10 μm) bacterioplankton vs. the concentration of colCHO (R2 = 0.3779, p = 0.0093). [file Image_1.TIF]

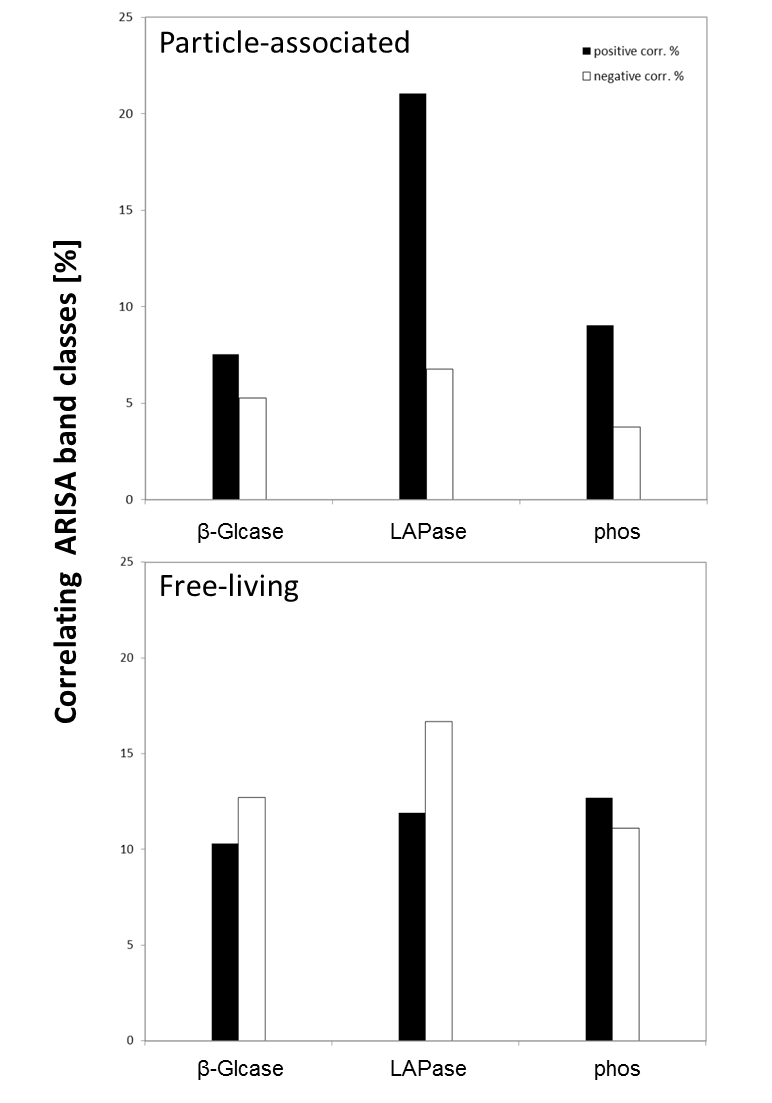

Supplement: FIGURE S2 — Percent of particle-associated and free-living ARISA band classes (fluorescence intensity) correlating (Spearman Rank Correlation; rs > 0.5, p < 0.05) to the maximum velocity (Vmax) of the extracellular enzymes β-glucosidase (β-Glcase), leucine aminopeptidase (LAPase) and phosphatase (phos) during spring 2010 at the Helgoland Roads sampling station. [file Image_2.tif]

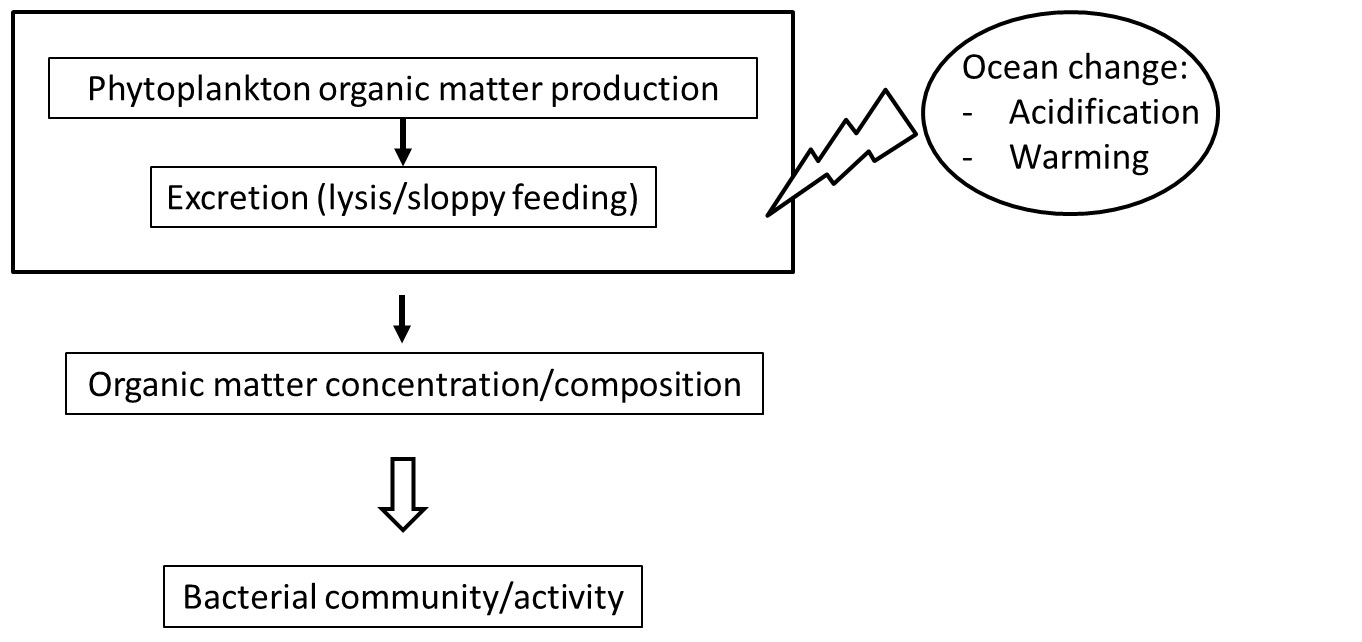

Supplement: FIGURE S3 — Schematic representation of a mechanism for secondary effects of ocean change on bacterioplankton community and activity. [file Image_3.TIF]
