# Supplementary material for: Combined Carbohydrates Support Rich Communities of Particle-Associated Marine Bacterioplankton
Source: Front Microbiol. 2017 Jan 31;8:65. doi: 10.3389/fmicb.2017.00065 (PMC5281597; doi:10.3389/fmicb.2017.00065)
Supplement: TABLE S1 — Analyzed carbohydrates and abbreviations. [file Table_1.docx]

Table S1

| **Abbreviation** | **Carbohydrate** |
| --- | --- |
|  |  |
| **Neutral sugars** |  |
| Ara | arabinose |
| Fuc | fucose |
| Gal | galactose |
| Glc | glucose |
| Man/Xyl | mannose/xylose |
| Rha | rhamnose |
|  |  |
| **Acidic sugars** |  |
| GalUA | galacturonic acid |
| GlcA | gluconic acid |
| GlcUA | glucuronic acid |
| Mur | muramic acid |
|  |  |
| **Amino sugars** |  |
| GalN | galactosamine |
| GlcN | glucosamine |

Table S2

Table S3

|  | **SAR11-411** | **ROS537** | **GAM42a** | **CF319a** |
| --- | --- | --- | --- | --- |
| **Abiotic** |  |  |  |  |
| temp | - | ø | ++ | ø |
| sal | ø | ø | ø | ø |
| pH | + | ø | ++ | ø |
| **Biotic** |  |  |  |  |
| SiO2 | + | ø | -- | - |
| PO4 | ø | ø | ø | ø |
| DIN | ø | ø | ø | -- |
| diatoms | ø | ø | ø | ø |
| Chl *a* | - | ø | ++ | + |
| POC | ø | ø | ++ | ø |
| **Carbohydrates** | |  |  |  |
| colCHO | ø | ø | ++ | ø |
| Ara | ø | ø | ø | ø |
| Man/Xyl | ø | ø | ø | ø |
| Glc-A | ø | ø | ø | ø |
| Mur-A | ø | ø | ø | - |
| Gal-URA | ø | ø | + | ø |
| **Extracellular enzymes** | |  |  |  |
| b-glu | - | ø | + | ø |
| LAPase | -- | ø | + | ø |
| phos | ø | ø | ++ | ø |
